# Analysis of Autistic Adolescents’ Essays Using Computer Techniques

**DOI:** 10.1007/s10803-024-06482-4

**Published:** 2024-07-27

**Authors:** Izabela Chojnicka, Aleksander Wawer

**Affiliations:** 1 Faculty of Psychology, University of Warsaw, Stawki 5/7, Warsaw, 00-183 Poland; 2 Institute of Computer Science, Polish Academy of Sciences, Warsaw, 01- 248 Poland

**Keywords:** Autism, Narrative abilities, Pragmatic language, Sentiment, Natural language processing, Computational models

## Abstract

**Purpose:**

Challenges associated with narrative discourse remain consistently observable across the entire spectrum of autism. We analyzed written narratives by autistic and non-autistic adolescents and aimed to investigate narrative writing using quantitative computational methods.

**Methods:**

We employed Natural Language Processing techniques to compare 333 essays from students in the final eighth grade of primary school: 195 written by autistic and 138 by non-autistic participants.

**Results:**

Autistic students used words with a positive emotional polarity statistically less frequently (*p* < .001), and their stories were less abstract (*p* < .001) than those written by peers from the non-autistic group. However, autistic adolescents wrote more complex stories in terms of readability than participants from the non-autistic group (*p* < .001). The writing competencies assessed by teachers did not differ significantly between the two groups.

**Conclusion:**

Findings suggest that written narratives by autistic individuals may exhibit characteristics similar to those detected by computational methods in spoken narratives. Collecting data from national exams and its potential usefulness in distinguishing autistic individuals could pave the way for future large-scale and cost-effective epidemiological studies on autism.

## Introduction

Autistic individuals comprise a very heterogeneous group regarding language capacity, ranging from non-speaking to fluently speaking individuals (World Health Organization, 2021). Challenges related to the pragmatic use of language are observed across the entire autism spectrum in individuals with varying levels of intellectual and linguistic abilities, including those individuals who display structural language skills within the typical range (Schaeffer et al., 2023). Difficulties with pragmatic language in autism include narrative discourse (Baixauli et al., 2016). Narrative skills develop from the early stages of life, being an important way of sharing experiences among children and a tool for understanding the world, as well as incorporating experiences into the sense of one’s self (Fivush et al., 2011). In this study, we use quantitative computational methods to investigate the narrative writing of autistic adolescents, which remains under-researched in comparison to spoken narrative abilities (Finnegan & Accardo, 2018).

Many previous studies on narration in autism have focused on examining spoken narratives, mainly storytelling. According to the meta-analysis by Baixauli et al. (2016), spoken narratives by autistic participants were shorter and less diverse (measured by the number of different words) than narratives produced by typically developing children and adolescents. In terms of narrative macrostructure, spoken narratives of autistic children were less causally connected and considerably less coherent than those of typically developing peers. Additionally, autistic individuals used significantly fewer internal state terms than neurotypical participants. Internal state language (ISL) refers to the vocabulary that describes character perceptions, emotions, and mental states. A study by Siller et al. (2014) revealed a relationship between the presence of ISL vocabulary in narratives and theory of mind. Based on the findings of the aforementioned meta-analysis (Baixauli et al., 2016), autistic individuals with higher intelligence levels encounter significant difficulties compared to their peers in verbally describing internal states. The authors argued that this might indicate an asynchrony in the development of cognitive and linguistic aspects with socioemotional meaning.

Yet, spoken discourse differs from written discourse. The former typically occurs in interaction, inherently involving reciprocity, the experience and perception of affect, and encompassing broad non-verbal and paralinguistic features. The latter may be more deliberate, allowing time to think through the narrative and revise it if the writer decides to do so. Writing, particularly handwriting, is a perceptual-motor activity that requires the coordination of multiple simultaneous tasks: the mechanics of writing, content, and organization (Finnegan & Accardo, 2018; Shevchuk-Hill et al., 2022). However, similar to spoken discourse, it may involve the theory of mind abilities, as the story gains if the writer is able to adopt the reader’s perspective. Some authors have analyzed narrative writing in autism (e.g., Baixauli et al., 2021; Price et al., 2019). Shevchuk-Hill et al. (2022) compared stories by autistic (*n* = 19) and non-autistic (*n* = 23) university students using automated methods. Authors concluded that writing may be a strength for autistic students whose stories were rated at a higher reading level, contained fewer grammatical errors, and had more positive writing affect (Shevchuk-Hill et al., 2022). In contrast, a study of autistic adolescents indicated difficulties with writing skills, specifically in terms of productivity, lexical diversity, and overall coherence of the story (Baixauli et al., 2021), similar to results reported for spoken narratives. Therefore, it seems that there is no clear consensus regarding written narrative skills in autism.

In this study, we aimed to build on prior research on narrative in autism by investigating the utility of natural language processing techniques (NLP) for quantifying the narrative abilities in the writing of autistic adolescents. According to our knowledge, this is the first study utilizing sentiment and linguistic abstraction analyses to examine narratives written by autistic individuals. Sentiment analysis extracts subjective information in terms of the positive and negative emotional tones of the text. With the development of social media, it has become a frequently utilized NLP technique (Denecke & Reichenpfader, 2023). Sentiment analysis considers various types of words, not only those that explicitly describe emotions (e.g., ‘happy’, ‘sadness’) but also other words that carry positive or negative connotations (e.g., ‘good’, ‘wise’, ‘home’; Wawer, 2019). Thus, sentiment analysis is broader than the analysis of words that refer to internal emotional states.

Linguistic abstraction was assessed using the linguistic category model (LCM), a model of interpersonal language that provides means to investigate linguistic devices used to represent social events (Semin, 2012). The LCM typology classifies words based on their degree of abstraction, with the principal distinction established among three categories of verbs: Descriptive Action Verbs (DAVs), Interpretative Action Verbs (IAVs), and State Verbs (SVs) (for the Polish version we used see Wawer & Sarzyńska, 2018). DAVs represent the most concrete verbs used to depict a singular and observable event (e.g., ‘A kicks B’). IAVs describe specific observable events (e.g., ‘A hurts B’), and they are more abstract than DAVs as they omit the perceptual aspects of an action. The most abstract category encompasses State Verbs, which pertain to mental states (e.g., ‘to think,’ ‘to understand’) and emotional states (e.g., ‘to admire’) or changes thereof. The model also considers adjectives (ADJ) as the abstract end of the continuum. Therefore, in addition to enabling the analysis of the level of abstraction, LCM also allows assessing the number of words describing the mental and emotional states of the person (Beukeboom et al., 2013).

Combining both methods, sentiment analysis and LCM, may be a useful automated alternative to analyzing the internal state language of narratives using hand-coding methods. However, it should be noted that the two methods do not analyze exactly the same words as the hand-coding approach to ISL. Additionally, we calculated a readability index as a lexical complexity metric.

We obtained essays written and evaluated in a standardized manner as part of a nationwide exam. It allowed for the comparison of autistic adolescents with peers from the population, including both neurotypical individuals and those with possible other neurodevelopmental and psychiatric conditions. In our previous research, where we employed NLP techniques and machine learning, we analyzed differences in spoken narratives between autistic individuals and typically developing peers (e.g., Wawer & Chojnicka, 2022). Here, we compared autistic adolescents with a more diverse group from the population. We hypothesized that stories written by autistic adolescents contain fewer words with emotional polarity and exhibit lower levels of linguistic abstraction than stories written by their peers, similar to differences detected for spoken narratives (Chojnicka & Wawer, 2020).

## Methods

### Data

The essays were written as part of the nationwide eighth-grade Polish language time-limited exam, conducted upon the completion of primary school education, simultaneously undertaken by all students throughout the country, under standardized conditions, and with standardized examination sheets. There were ten versions of exam sheets tailored to accommodate the special needs of some students: (1) for students without disabilities and those with specific learning disorders (OPOP-100); (2) for students with autism, including Asperger’s syndrome (OPOP-200); (3) for students with visual impairments font size 16 pt (OPOP-400); (4) for students with visual impairments font size 24 pt (OPOP-500); (5) for deaf and hard of hearing students (OPOP-700); (6) for students with mild intellectual disability (OPOP-800); (7) for students with aphasia (developmental language disorder; OPOP-900); (8) for students with motor disabilities caused by cerebral palsy (OPOP-Q00); (9) for students whose limited knowledge of Polish makes it difficult to understand the text being read (OPOP-C00); (10) for students who are citizens of Ukraine (OPOU-C00). Students who used type (2) exam sheets, based on a clinical diagnosis of Autism Spectrum Disorder confirmed by a psychiatrist and a commission responsible for making decisions regarding the need for special education, were assigned to the Autistic Group. The Non-autistic Group comprised students who took exams on type (1) sheets.

One of the tasks on the exam was to handwrite an essay on a given topic. Autistic students were allowed to opt out of handwriting and type their essays in case of handwriting legibility issues. In this paper, we use the term *essay* as a written composition. The students chose one of two types of essays: an opinion essay or a short story. The instruction for the task was: “*Choose one of the given topics and write an essay. • Remember to maintain the discourse style indicated in the topic: write an opinion essay or a short story. • In your essay*,* refer to the selected compulsory reading. The list of compulsory readings is on page 3 of this examination paper. • Your work should be at least 200 words long. • Write the essay in the designated place. Do not write in the margin.*” We only analyzed essays of the short story type. In the section designated for students from the general population (OPOP-100), the topic for the story was: “*Write a story about meeting one of the characters from the selected compulsory reading. A shared adventure prompted you to reflect that it was worth moving to the world depicted in this reading. The essay should demonstrate that you are familiar with the selected compulsory reading.*” In the section designated for students within the autism spectrum (OPOP-200), the topic was formulated slightly differently: “*Imagine you have the opportunity to travel back in time to the world of one of the compulsory readings. Write a story about your adventure in this world. The essay should demonstrate that you are familiar with the selected compulsory reading.*”

As part of the exam, stories written by students were subject to assessment conducted by teachers trained to follow standardized examination guidelines. It included the following categories: Topic Development, Creative Elements, Literary Skills, Text Composition, Style, Language, Spelling, and Punctuation. The total score for the task was determined by summing the points in each category.

We received data from the District Examination Boards responsible for storing documentation from the nationwide eighth-grade examination. We asked the Examination Boards to select essays in a way that there were roughly equal proportions of students who achieved high and low exam results in both groups, with a majority of students obtaining average scores; apart from that, sampling was conducted in a random fashion. Anonymized scans of the essays, presented as .pdf files, were provided, accompanied by information on each student’s sex, year of birth, and ASD diagnosis (or lack thereof). Optical character recognition for converting scans into text was not effective for our data. Therefore, the essays were manually transcribed by two trained students into .txt files, preserving the original spelling, paragraph structure, punctuation, and other aspects such as capitalization while omitting portions of the text that were crossed out by a student. A third person (one of the authors, IC) revised 30% of the prepared transcripts.

### Participants

We collected 333 stories from students in the final, eighth grade of primary school: 195 stories written by autistic students (Autistic Group; average age 14.85 years; 25% girls); and 138 written by students from the general population (Non-autistic Group; average age 14.64 years; 25% girls). The latter included neurotypical adolescents as well as, most likely, undiagnosed adolescents with neurodevelopmental or psychiatric conditions present in the population. The Non-autistic Group specifically included adolescents with developmental learning disorders (often referred to as dyslexia, dysorthographia, or dyscalculia), as students with developmental learning disorders took the exams on the same exam sheets as students from the general population. Despite having a similar average age, the groups exhibited statistically significant differences (*p* = .006; Table 1). Within the Autistic Group, some students were 16 (*n* = 18) and 17 years old (*n* = 1). The Non-autistic Group consisted solely of students aged 14 and 15.

**Table 1:** Characteristics of the sample

	Autistic Group (*n* = 195)	Non-autistic Group (*n* = 138)	*p*-value
Girls %	25	25	0.955
Chronological age in years *M (SD)*	14.85 (0.64)	14.64 (0.48)	0.006
Story total score assigned by a teacher	11.28 (4.27)	11.54 (4.14)	0.599
Number of words in a story *M (SD)*	369.49 (135.56)	415.48 (145.46)	0.007

### Sentiment Analysis, Linguistic Abstraction, and Readability

We employed a manually created dictionary of 5421 positive and negative words, representing the sentiment of the most frequent sense of a word and therefore requiring no word sense disambiguation. The level of language abstraction was calculated according to the weighted summation formula: DAV + IAV*2 + SV*3 + ADJ*4. Applied computational methods are described in Chojnicka & Wawer (2020). To evaluate the readability of an essay, we calculated the Gunning Fog Index (Gunning, 1952), which estimates the years of formal education necessary for a person to comprehend the text upon their initial reading. We used the textstat library^1^ to perform the computations.

## Results

### Essay Length and Scores

The essays did not differ significantly between the two groups regarding the linguistic and literary categories, and the total score assigned by teachers. Therefore, it appears that the writing competencies assessed in a school-oriented manner by teachers were similar in both groups. We also assessed whether the number of tokens (words) differentiates both groups. Essays by autistic adolescents were shorter compared to those by peers from the control group (*U* = 11108.50, *Z* = -2.711, *p* = .007, *r* = -.149; Table 2). Since a statistically significant difference was detected, we carried out subsequent dictionary-based analyses (sentiment, LCM) using normalized values, wherein each parameter was divided by the number of tokens.

**Table 2:** Means (and SD) for the number of words in a given category in an essay

	Autistic Group M (SD)	Non-autistic Group M (SD)	*p*-value
Positive words	11.74 (7.38)	17.42 (9.19)	< 0.001
Negative words	7.87 (5.22)	8.70 (5.09)	0.857
DAVs	22.18 (10.52)	25.69 (11.03)	0.151
IAVs	14.53 (7.14)	17.25 (8.38)	0.142
SVs	13.43 (6.56)	17.20 (8.46)	0.001
ADJs	1.69 (1.71)	2.33 (1.88)	0.028
LCM level	98.28 (40.00)	121.09 (48.00)	< 0.001
Gunning Fog Index	11.72 (15.99)	7.93 (1.99)	< 0.001

In analyses, we used normalized values, wherein each parameter was divided by the number of tokens. The p-values included in the table refer to these analyses. However, here, we included the sum of words without normalization to ensure clarity for the reader. Values for the Gunning fog index are reported here without excluding the outliers from the Autistic Group

### Sentiment and Language Abstraction Analyses

Autistic students used words with a positive evaluative meaning statistically less frequently in their stories compared to participants from the Non-autistic Group (*U* = 8611.00, *Z* = -5.597, *p* < .001, *r* = -.307). We found no such difference for words with a negative evaluative meaning (*p* = .857).

We observed statistically significant differences for mental and emotional State Verbs (SVs), Adjectives (ADJs), and the overall level of Language Abstraction. Autistic students used SVs (*U* = 10655.5, *Z* = -3.235, *p* = .001, *r* = -.177) and ADJs (*U* = 11009.50, *Z* = -2.826, *p* = .028, *r* = -.155) less frequently in their essays than peers from the control group (Table 2). The calculated language abstraction for the entire essays was lower in the ASD in comparison to the Non-Autistic Group (*U* = 9666.50, *Z* = -4.378, *p* < .001, *r* = -.240). We did not find statistically significant differences for less abstract verb categories.

### Essays’ Readability

To evaluate the readability of an essay, we calculated the Gunning Fog Index, obtaining statistically significant differences (*U* = 10129.50, *Z* = -3.843, *p* < .001, *r* = -.211). Then, we excluded three outliers from the Autistic Group who scored significantly higher than all other participants in the study, indicating the use of an unusually difficult and complex language. After removing the outliers, the differences remained statistically significant (*U* = 10129.50, *Z* = -3.648, *p* < .001, *r* = -.200). Autistic students wrote more complex stories in terms of readability (*M* = 9.85, *SD* = 5.49) than peers from the Non-autistic Group (*M* = 7.93, *SD* = 1.99, Table 2).

## Discussion

Previous studies on narrative skills in autism have primarily focused on spoken narratives. In this study, we present results pertaining to written narrations, offering a novel perspective in several ways: (1) written narrations by autistic individuals exhibit similarities in certain characteristics to spoken narrations, (2) these characteristics can be detected using automated, quantifiable, and objective computer measures, and (3) we utilized data from standardized nationwide exams, enabling the study of large groups.

Previous studies on internal state language in autism have mostly focused on verbs and adjectives relating to characters’ emotional (e.g., ‘laugh’, ‘happy’) and cognitive states (e.g., ‘know’, ‘confused’). The results of the meta-analysis conducted by Baixauli et al. (2016) indicate that autistic children use fewer ISL terms than neurotypical peers in spoken narratives. In our study, written narratives by autistic adolescents were similar to spoken narratives in terms of sentiment and the level of linguistic abstraction. In the stories, students from both groups included more words with a positive emotional tone than words with a negative emotional tone. However, autistic students used fewer words with positive emotional polarity than students from the Non-autistic Group with a moderate effect size. This finding aligns with our previous research on spoken narratives produced during the ADOS-2 (Lord et al., 2012) Picture Book task (Chojnicka & Wawer, 2020). The results we have obtained using the same NLP techniques (sentiment analysis and Linguistic Category Model) suggest that autistic individuals not only use fewer direct expressions and descriptions of positive emotions but also fewer other emotionally positively related words (such as ‘trust’, ‘home’).

Another result consistent with our prior research on spoken narratives is the lower level of abstraction of the essays of autistic adolescents. We found statistically significant differences in the overall level of an essay’s abstraction, the number of LCM Adjectives, and State Verbs related to mental and emotional states or changes therein. Autistic students used fewer Adjectives and State Verbs, and their essays were less abstract than those written by non-autistic peers. The current results indicate that the narratives of autistic students were more concrete, consisting primarily of direct (e.g., “They built a sandcastle on the beach”) rather than subjective descriptions related to cognitive and emotional states (e.g., “They knew that honesty was the best option”). In the article on spoken narratives produced by autistic individuals during the ADOS-2 Picture Book task (Chojnicka & Wawer, 2020), we demonstrated less frequent use of DAVs and SVs, as well as a lower overall level of abstraction in the Autistic Group.

In our previous studies (Chojnicka & Wawer, 2020; Wawer & Chojnicka, 2022) on spoken narratives, participants in the autistic and comparison groups were matched for age, sex, and non-verbal and verbal intelligence quotients. In the current project, we did not have access to data regarding the intellectual functioning of the participants. However, the groups did not differ significantly in writing skills measured by the number of points awarded by teacher-examiners in the categories: *Topic Development*,* Creative Elements*,* Literary Skills*,* Style*,* Language*,* Text Composition*,* Spelling*,* Punctuation*, and the Total score. Nonetheless, autistic students wrote shorter essays than non-autistic peers. This aligns with previous studies pointing to lower productivity of spoken narratives from autistic individuals (Baixauli et al., 2016).

To assess the readability of an essay, we computed the Gunning Fog Index (Gunning, 1952), which estimates the years of formal education necessary for an individual to comprehend the text upon their initial reading. The higher the index value for a given text, the more years of education are needed for complete understanding. The Gunning Fog Index takes into account the length of sentences and the number of “complex” words with four or more syllables (for the Polish language; in English, words with three syllables or more are considered complex words; Świeczkowski & Kułacz, 2021). The outcomes of our study suggest that autistic adolescents wrote more complex essays in terms of readability than non-autistic peers. This aligns with the results obtained in the study of written stories by university students (Shevchuk-Hill et al., 2022). The authors used the Dale-Chall readability formula and suggested a higher reading level for stories written by autistic young adults. This result may be related to the tendency of some autistic individuals to use pedantic, encyclopedic vocabulary and overly formal and adult-like language (Luyster et al., 2022). An interesting finding, despite higher essay complexity as measured by the number of syllables, is that people with autism produced less abstract essays as measured by the LCM. The connection between LCM and the Gunning-Fog index is not explored, examining it, especially in the case of morphologically rich languages such as Polish, ​​is an interesting subject of future research.

However, the obtained results should be treated with caution as the Autistic Group included several older students than the Non-autistic group. In Poland, finishing primary school at a later age often results from postponing compulsory schooling, usually by a year, in the case of children considered not developmentally ready to start school, which might be the case for autistic students. It may also result from not being promoted to the next class due to insufficient mastery of the educational material at a given level. However, in both cases, all students completed the same number of years of formal education.

We would like to address the limitations of the current study that suggest directions for future research. The strength of the study was the relatively large number of collected essays, but we had limited clinical characteristics of participants, and as discussed earlier, we did not have data on IQ scores. The second limitation was the slightly different task instructions received by participants from the two groups. One aspect that draws attention is that the instruction for the Non-autistic Group included the phrase “shared adventure”, whereas the instruction for the Autistic Group used the wording “your adventure”. The first implies interpersonal experiences, while the second does not. It could have an impact on the results we obtained. However, analyses involving human raters indicate that both instructions did not differentiate the essays in an evident manner. In another project using the same data, human raters, psychologists experienced in autism diagnosis, were tasked with assigning participants to groups after reading their stories. The raters’ effectiveness was close to random (unpublished data). Hence, the essays from both groups did not differ in a way that would be noticed by human raters.

Another limitation is related to the assumptions underlying the computational methods used. The meaning of certain words, as they occur in the text, depends on their neighboring words. This could be captured using an approach based on Construction Grammar (Goldberg, 1995, 2006). When applied to dialogic engagement, using the notions of lexical and syntactic resonance, Tantucci and Wang (2023) identified differences in creativity and intersubjective engagement in children with ASD in contrast with the neurotypical population. However, in our approach, the measures of sentiment and the LCM model use the most frequent sense of a word. We argue that this is a strong baseline approach, outperforming automated word sense disambiguation tools available in Polish. Rare senses and usages contribute less to the overall error. Moreover, this contribution is unlikely to have a significant influence on comparisons between groups that we make. On the one hand, the advantage of our approach is full automation and no annotation inconsistencies introduced by inter-annotator disagreements. On the other hand, the method proposed by Tantucci and Wang (2023) offers a more fine-grained analysis, and better handling of word senses.

We would like to draw the reader’s attention to another aspect. Most of the essays were handwritten, but in the case of the Autistic Group, there were also essays written using a keyboard. This is a technical difference between the groups that may also affect the written stories, although we do not propose a hypothesis regarding the direction of this influence.

Our study indicates that automated, quantitative computational methods are effective means for analyzing written narratives in autism. A significant conclusion drawn from our study is that written narratives by autistic individuals proved similar to spoken narratives in terms of characteristics detected by computational methods. Autistic adolescents used fewer words with positive emotional polarity and verbs describing the mental and emotional states of the characters, and their essays were less abstract but more complex in terms of readability than the narratives of non-autistic peers. Collecting data from national exams and its potential usefulness in distinguishing autistic individuals could pave the way for future large-scale and cost-effective epidemiological studies on autism.

## Data Availability

Data is available upon request from the corresponding author.

## Footnotes

1 https://pypi.org/project/textstat/.
